# SARS-CoV-2 Modulation of HIV Latency Reversal in a Myeloid Cell Line: Direct and Bystander Effects

**DOI:** 10.3390/v16081310

**Published:** 2024-08-17

**Authors:** Patricio Jarmoluk, Franco Agustín Sviercz, Cintia Cevallos, Rosa Nicole Freiberger, Cynthia Alicia López, Guido Poli, M. Victoria Delpino, Jorge Quarleri

**Affiliations:** 1 Consejo Nacional de Investigaciones Científicas y Tecnológicas (CONICET), Instituto de Investigaciones Biomédicas en Retrovirus y Sida (INBIRS), Laboratorio de Inmunopatología Viral, Universidad de Buenos Aires (UBA), Buenos Aires C1121ABG, Argentina; patriciojarmoluk@gmail.com (P.J.); francosviercz@gmail.com (F.A.S.); cevalloscintia@gmail.com (C.C.); freibergernicole@gmail.com (R.N.F.); alilopez1996@gmail.com (C.A.L.); mdelpino@ffyb.uba.ar (M.V.D.); 2 Division of Immunology, Transplantation and Infectious Diseases, IRCCS San Raffaele Scientific Institute, 20132 Milan, Italy; poli.guido@hsr.it; 3 School of Medicine, Vita-Salute San Raffaele University, 20132 Milan, Italy

**Keywords:** HIV, latency, U1, SARS-CoV-2, COVID-19, macrophage polarization

## Abstract

Coronavirus disease 2019 (COVID-19) might impact disease progression in people living with HIV (PLWH), including those on effective combination antiretroviral therapy (cART). These individuals often experience chronic conditions characterized by proviral latency or low-level viral replication in CD4+ memory T cells and tissue macrophages. Pro-inflammatory cytokines, such as TNF-α, IL-1β, IL-6, and IFN-γ, can reactivate provirus expression in both primary cells and cell lines. These cytokines are often elevated in individuals infected with SARS-CoV-2, the virus causing COVID-19. However, it is still unknown whether SARS-CoV-2 can modulate HIV reactivation in infected cells. Here, we report that exposure of the chronically HIV-1-infected myeloid cell line U1 to two different SARS-CoV-2 viral isolates (ancestral and BA.5) reversed its latent state after 24 h. We also observed that SARS-CoV-2 exposure of human primary monocyte-derived macrophages (MDM) initially drove their polarization towards an M1 phenotype, which shifted towards M2 over time. This effect was associated with soluble factors released during the initial M1 polarization phase that reactivated HIV production in U1 cells, like MDM stimulated with the TLR agonist resiquimod. Our study suggests that SARS-CoV-2-induced systemic inflammation and interaction with macrophages could influence proviral HIV-1 latency in myeloid cells in PLWH.

## 1. Introduction

People living with HIV (PLWH) are considered a vulnerable group with an elevated susceptibility to coronavirus disease 2019 (COVID-19), an infection of pandemic proportions caused by Severe Acute Respiratory Syndrome (SARS) Coronavirus 2 (SARS-CoV-2) that has been curtailed mostly because of the extraordinary efficacy of mRNA-based vaccines [1]. In sharp contrast, no vaccines have been yet discovered for the prevention of HIV-1 infection. Combination antiretroviral therapy (cART) not only blocks disease progression while promoting immunologic reconstitution but can also be effectively used as a preventative tool as pre-exposure prophylaxis [2]. A second major obstacle to the achievement of an HIV-1 cure is the existence of a heterogeneous reservoir of infected cells not eliminated or curtailed by cART [3,4]. In addition to the well-established role of “resting memory” and related subsets of CD4+ T lymphocytes, HIV-1 can also infect circulating monocytes in vivo, which can irreversibly differentiate into macrophages as monocyte-derived macrophages (MDMs) upon crossing the blood–tissue barrier. These HIV-infected MDMs, together with tissue resident macrophages (TRMs) frequently independent from the bone marrow in their ontogeny, are more resistant to the cytopathic effects of HIV and significantly contribute to the latent viral reservoir [5] and are considered a major source of residual replication-competent viremia during ART [6]. Macrophage-associated HIV reservoirs has been demonstrated in various organs and tissues, including lung alveolar macrophages [7,8,9,10].

There is limited knowledge on whether SARS-CoV-2 infection may perturb, directly or indirectly, the HIV reservoir in PLWH, although it is well established that several pro-inflammatory cytokines, strongly induced by SARS-CoV-2 infection, can lead to the reversal of proviral HIV-1 latency in both primary and immortalized T lymphocytic and myeloid cells [11]. In this regard, SARS-CoV-2 infection may lead to the reactivation of microbial pathogens, including persistent herpesviruses such as Epstein–Barr virus (EBV), HHV-6, and cytomegalovirus (CMV) [12,13,14]. In addition, infection of SARS-CoV-2 may alter the HIV reservoir size among PLWH; as well, the Pfizer/BioNTech BNT162b2 SARS-CoV-2 mRNA vaccine has been shown to induce HIV reactivation in PBMCs of SARS-CoV-2-naïve, ART-treated individuals with HIV ex vivo via innate immune sensing of mRNA and subsequent activation of NF-κB [15,16].

One of the typical features of SARS-CoV-2 infection is indeed the heightened inflammatory reaction observed in severe COVID-19 cases, which leads to an excessive secretion of pro-inflammatory cytokines commonly referred to as a “cytokine storm”. In this regard, macrophages play a crucial role in both triggering and mitigating systemic inflammation as they swiftly generate pro-inflammatory and regulatory cytokines in response to local inflammation and pathogenic invasion [17]. Macrophages exposed to or infected with SARS-CoV-2 have been reported to exhibit a dominant pro-inflammatory “M1” polarized profile, thus contributing to the cytokine storm. High levels of SARS-CoV-2 RNA can act as a pathogen-associated molecular pattern (PAMP), leading to macrophage hyperactivity, excessive inflammatory damage, and tissue fibrosis [18]. Macrophage-mediated clearance of SARS-CoV-2-infected cells could be associated with the secretion of pro-inflammatory cytokines, such as IL-6 and TNF-α, leading to a strong activation of plasmacytoid dendritic cells (pDCs) secreting high levels of IFN-α and TNF-α [19]. In this regard, the alveolar space of patients with SARS-CoV-2 pneumonia is significantly enriched with activated CD4^+^ and CD8^+^ T cells and monocytes; T cells release IFN-γ, enhancing the production of other inflammatory cytokines from alveolar macrophages fueling sustained alveolar inflammation [20]. If this scenario occurs in PLWH, it is plausible that SARS-CoV-2-induced alveolitis may lead to perturbations of proviral latency in local CD4+ T cells or monocyte/macrophages.

Using a well-established myeloid in vitro cell model of reversible HIV latency, i.e., the chronically HIV-1-infected U1 cell line derived from U937 promonocytic cells [21], we investigated whether SARS-CoV-2 can lead to proviral reactivation either through direct interaction or indirectly via cytokines released by human MDMs exposed to SARS-CoV-2. We observed that proviral reactivation indeed occurred in U1 cells after incubation with soluble mediators released by MDMs exposed to SARS-CoV-2, and, to a lesser extent, by a direct interaction of latently infected cells with SARS-CoV-2.

## 2. Materials and Methods

### 2.1. Cell Lines, Primary Cells, and Reagents

Myeloid U1 cells used in this study were originally obtained from the AIDS reagent program of the National Health Institute of the United States of America (NIH, Stapleton, NY, USA) [22,23]. U1 cells were originally cloned from promonocytic U937 cells surviving acute infection with the HIV-1 LAI/IIIB strain [24]. Cells were maintained in RPMI 1640 medium (Gibco, New York, NY, USA) supplemented with 10% fetal bovine serum (FBS, Sigma Aldrich, Buenos Aires, Argentina) in an incubator at 37 °C and 5% CO_2_ atmosphere. The uninfected counterpart, i.e., the U937 cell line, was used in some experiments. The cells were routinely tested for mycoplasma contamination using the MycoAlert^®^ Mycoplasma detection kit (LT07-318, Lonza, Tampa, FL, USA).

U1 cells were seeded at 10^5^ cells/mL in 24-well plates and separated into three experimental groups:

1. the unstimulated group that was not exposed to reactivating stimuli (negative control);
2. the stimulated group incubated with phorbol 12-myristate 13-acetate (PMA; P8139, Merck, St. Louis, MO, USA) at 30 ng/mL to induce proviral reactivation from latency (positive control);
3. the study group exposed to different candidate stimuli.

After 4 h of incubation in the presence or absence of the different stimuli, cells were centrifuged at 300× *g* for 4 min and resuspended in 100 μL of phosphate-buffered saline (PBS). HIV reactivation in U1 cells was monitored by measuring HIV-1 p24 Gag expression using a Cytek^®^ Northern LightsTM 3000 flow cytometer (Cytek Biosciences Inc., Fremont, CA, USA). Briefly, cells were fixed and permeabilized with a BD Cyto Fix/Perm kit (BD Biosciences, San Jose, CA, USA), washed with PBS containing 1% FBS, and stained with anti-HIV-1 p24 Gag phycoerythrin (PE) mAb KC57 (Beckman Coulter, Brea, CA, USA) diluted at 1:250. Isotype-matched mAbs were used as negative controls.

Human primary monocytes were obtained after a Percoll gradient from peripheral venous blood of anonymous healthy donors and differentiated as monocyte-derived macrophages (MDMs) by adhesion to plastic flasks, as described previously [25,26]. After overnight incubation, non-adherent cells were removed by three washes with warm phosphate-buffered saline (PBS). The purity of monocytes was ≥90%, as determined by immunofluorescent staining with anti-CD14 monoclonal antibody ([mAb] BD Pharmingen, San Diego, CA, USA) using a Cytek^®^ Northern Lights 3000™ Full Spectrum Flow Cytometer. Then, monocytes were seeded in 24-well plastic plates at 5 × 10^5^ cells/mL in RPMI medium supplemented with 10% FBS, 2 mM of L-glutamine (Gibco), 1 mM of sodium pyruvate (Gibco), penicillin-streptomycin (Sigma-Aldrich, Burlington, MA, USA), and Macrophage Colony-Stimulating Factor (M-CSF) (10 ng/mL) (StemCell Technologies, Vancouver, BC, Canada) for 6 days. On day 6, the differentiated cells were collected and verified to be ≥90% CD68+ by flow cytometry. Human MDMs were either left unstimulated (M0 macrophages) or were further stimulated for 24 h to induce their functional polarization. To obtain M1 cells, MDMs were incubated with ultrapure lipopolysaccharide (LPS, 100 ng/mL; InvivoGen, San Diego, CA, USA) and IFN-γ (20 ng/mL; from R&D, Minneapolis, MN, USA), whereas for M2 polarization, cells were incubated with IL-4 (20 ng/mL; R&D). The viability of unpolarized and polarized human MDMs was analyzed by flow cytometry (see below).

In some experiments, human MDMs were stimulated with the TLR 7/8 agonist Resiquimod (R-848, Alexis, Lausen, Switzerland) at 37 °C, 5% CO_2_ for 24 h. Then, the culture supernatant was collected and kept at −80 °C until testing on the U1 cell line as a conditioned medium (CM).

### 2.2. SARS-CoV-2 Variants, Viral RNA Quantification, and MDM Infection

The ancestral strain of SARS-CoV-2 (Wh) was contributed by Dr. Sandra Gallego from Universidad Nacional de Córdoba, Argentina, whereas the Omicron (BA.5) strain was sourced from a nasopharyngeal swab. Both strains were subsequently characterized, propagated, and titrated (at a concentration of 2.85 × 10^6^ TCID50 per mL) in Vero cells, a cell line obtained from African green monkey kidney. Vero E6 cells (ATCC, Rockville, MD, USA) were cultivated as monolayers in a 5% CO_2_ atmosphere at 37 °C in DMEM (Sigma-Aldrich, Buenos Aires, Argentina) supplemented with 2 mM L-glutamine, 10% FBS, 100 U/mL penicillin, and 100 μg/mL streptomycin.

SARS-CoV-2 genomic RNA was detected and quantified using Chemagic™ Viral DNA/RNA kit special H96 on the automated Chemagic™ 360 instrument (PerkinElmer, Rodgau, Germany). RNA was quantified using a NanoDrop™ (Thermo Scientific, San Diego, CA, USA) and was normalized before SARS-CoV-2 RNA detection by RT-qPCR (DisCoVery SARS-CoV-2 RT-PCR Detection Kit Rox, AP Biotech, Buenos Aires, Argentina) amplifying ORF1ab and N viral genes following the manufacturer’s instructions. In culture supernatants, viral load was calculated by interpolation of the corresponding Ct value with a standard curve, which had been built with the Ct values obtained following PCR amplification of samples containing serial dilutions of quantified SARS-CoV-2 positive RNA control (GISAID EPI_ISL_420600).

Human MDMs (5 × 10^5^ cells/mL) were cultured in RPMI supplemented with M-CSF (10 ng/mL; StemCell Technologies)—complete medium—for 3 days. Subsequently, they were incubated with SARS-CoV-2 (ancestral variant or Wh, MOI = 0.1) in RPMI without FBS for 4 h followed by 4–5 washes with PBS 1×. After 24, 48, or 72 h, their culture supernatants were collected. These conditions were operationally defined as “short term” SARS-CoV-2 exposure. Alternatively, after washing, MDMs (cultured in complete media) were re-exposed to SARS-CoV-2 for an additional 24, 48, or 72 h, collecting their culture supernatant; these conditions were defined as “long term” SARS-CoV-2 exposure. Conditioned media (CM) were obtained by centrifugation of cell culture supernatants at 3000× *g* for 10 min at 4 °C and stored at −80 °C until used. The ratio of CM and fresh completed medium for culturing the cells was 1:1 except for serial dilutions carried out with M1 and M2 supernatants, as indicated.

U1 cells and uninfected U937 (5 × 10^5^ cells/mL) were shortly exposed to SARS-CoV-2 (4 h) and allowed to rest for 72 h, or for extended times (24, 48, and 72 h). HIV latency reversal was evaluated by measuring intracellular p24 capsid antigen expression by flow cytometry, as described above.

### 2.3. SARS-CoV-2 Inactivation via UVC Irradiation

A UVC light tube (253.7 λ, 500 μW/cm^2^) was positioned 30 cm above the SARS-CoV-2 (5 mL, 5 × 10^4^ TCID 50/mL) virus culture plates (10 cm dishes) and exposed to UVC for 60 s for inactivation experiments. Post-irradiation, the SARS-CoV-2 was titrated using a TCID_50_ assay. Briefly, Vero E6 cells (2 × 10^4^ per well) in a 96-well plate were infected with 100 μL of ten-fold serially diluted virus-containing medium, with 8 replicates per dilution, and incubated at 37 °C for 3 days. Viral infection titer was measured by observing and quantifying the virus-induced cytopathic effect (CPE) [27].

### 2.4. Flow cytometry Analysis

MDMs were detached from plastic plates using Accutase^®^ (StemCell Technologies) to preserve surface antigens, followed by 30 min staining at 4 °C. Their activation profiles were assessed using monoclonal Abs against human CD80 (PE) (Biolegend, London, UK) and CD206 (APC) (Biolegend). A rabbit anti-human ACE2 primary polyclonal Ab (ab272690, Abcam, Cambridge, UK) and goat anti-rabbit IgG secondary (PE) (Abcam, UK) were used for ACE2 quantification. Cell death was evaluated with Ghost Dye Violet450 (Tonbo, Cytek Bioscienes Inc., Fremont, CA, USA) with cells exposed to freeze-thaw cycles as the positive control (Supplementary Figure S1). Data acquisition was performed on a Cytek^®^ Northern Lights 3000™ Full Spectrum Flow Cytometer (Cytek Biosciences Inc.) and analyzed with FlowJo.v10.6.2 (BD, Ashland, OR, USA).

### 2.5. ELISA for TNF-α, IL-6, and IL-1β Detection

TNF-α, IL-6, and IL-1β were measured in CM by sandwich ELISA using paired cytokine-specific mAbs, according to the manufacturer’s instructions (BD Pharmingen, USA).

### 2.6. TNF-α Neutralization

CM from uninfected M1-MDMs as well as from SARS-CoV-2-exposed MDMs (2.5 × 10^5^) were incubated for 2 h at 37 °C with Infliximab Chimeric Recombinant Human Monoclonal Antibody cA2 (Infliximab) at 25 μg/mL, a soluble TNF-α-neutralizing antibody (Cat#MA5-47798, ThermoFisher, Waltham, MA USA), before U1 cell incubation. The concentration of mAb used to neutralize TNF-α was determined by stimulating MDMs with recombinant TNF-α (1 ng/mL) in the presence of different concentrations of the mAb. As control, U1 cells were stimulated with human recombinant TNF-α (1 ng/mL); each experiment was repeated at least twice.

### 2.7. Statistical Analysis

The exact number of replicates per each experiment is specified in the figure legends. For datasets with a normal distribution (confirmed by the Kolmogorov–Smirnov test), two-tailed paired or unpaired t-tests were employed. Alternatively, two-tailed Mann–Whitney (unpaired test) or Wilcoxon matched-paired signed rank tests were applied using GraphPad Prism 7.0 (GraphPad Software Inc., Boston, MA, USA). Significance: * *p* ≤ 0.05; ** *p* ≤ 0.01; *** *p* ≤ 0.001, **** *p* ≤ 0.0001.

All experiments adhered to BSL-3 laboratory standards at INBIRS, with biological materials autoclaved and incinerated following institutional rules.

### 2.8. Ethical Approval

Ethical approval for this study was granted by the institutional review board and local ethical committee (Number: RESCD-2023-872). Buffy coats from healthy donors, aged 18 to 60 with a balanced gender ratio, were sourced from Hospital de Clínicas “José de San Martín”, Facultad de Medicina, Universidad de Buenos Aires. All human samples, obtained regardless of this study, were provided without personally identifiable information.

## 3. Results

### 3.1. Modulation of ACE2 Surface Expression in Uninfected Cells and in Latently HIV-Infected U1 Cells

Most immune cells in steady-state human peripheral blood samples, including freshly isolated CD14^+^ monocytes, poorly express ACE2, the SARS-CoV-2 entry receptor, on their surface. Then, when monocytes differentiate into alveolar macrophages after migrating into pulmonary tissues in response to inflammatory signals, mixing with local TRM, an upregulation of ACE2 expression has been reported [28].

**Figure 1:**
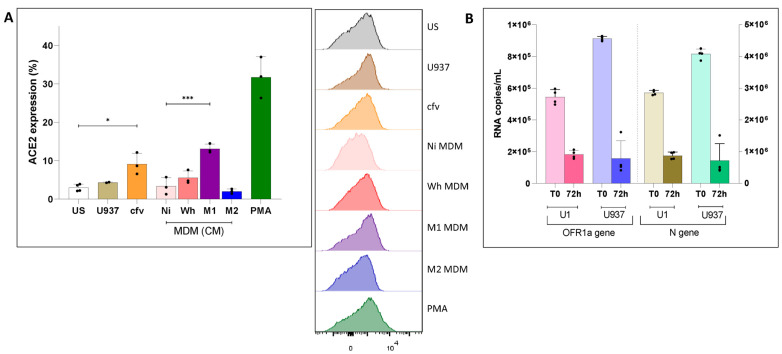
(**A**) Relative ACE2 expression levels in different U1 and U937 cells (**left**) and representative histograms of median fluorescence intensity (MFI) for each condition (**right**). Among U1, different conditions were evaluated, such as US: unstimulated U1; cfv: U1 exposed to SARS-CoV-2 cell-free; U937: unstimulated U937. U1 exposed to conditioned media (CM) obtained from non-infected MDM (Ni), MDM exposed to SARS-CoV-2 (Wh), MDM polarized (M1, or M2). PMA (Phorbol 12-myristate 13-acetate): positive control for reversal on both HIV-latently infected cell models. Data show the mean ± SEM, and statistical significance was calculated by one-way ANOVA. (**B**) Kinetics of the SARS-CoV-2 (Wh variant, MOI: 0.1) replication in myeloid (U1, U937) cells measured using RT-qPCR targeted to N and ORF1a genes in culture supernatant, as described in M&M. T0: supernatant obtained after three cell washes. 72 h: 3 days post-infection. Data are expressed as mean ± SD obtained from 4 independent experiments. (* *p* < 0.05; *** *p* < 0.001).

We observed that only a fraction of unstimulated (“US”) U1 cells expressed detectable levels of ACE2, as determined by flow cytometry, as shown in Figure 1 (2.9 ± 0.9%), as their uninfected U937 counterpart (4.2 ± 0.1%). ACE2 expression levels remained unchanged after incubation for 48 h with CM collected from different sources such as 3 days-old uninfected MDMs (“MDM-NI”, 3.3 ± 2.2%), SARS-CoV-2-exposed MDMs (“MDM-Wh”, 5.6 ± 1.8%), or M2-polarized MDMs (“MDM-M2”, 2.0 ± 0.6%). In contrast, U1 cells exhibited a significant increase in ACE2 expression (13.1 ± 1.2%) after incubation with CM from M1-polarized MDMs (“MDM-M1”) or when exposed to cell-free SARS-CoV-2 (“cfv”) Wh variant (9.1 ± 2.8%) (Figure 1). PMA-stimulated U1 cells exhibited the greatest increase (31.7 ± 5.3%) in cells expressing ACE2 (Figure 1A).

We have previously reported that human MDMs do not support productive infection by the SARS-CoV-2 ancestral variant [25]. To explore whether any of these cell lines could support productive SARS-CoV-2 infection, U1 and U937 cells were incubated with the Wh viral isolate (MOI = 0.1), and the levels of viral RNA in culture supernatants were measured by RT-qPCR for both the N and ORF-1a genes. However, the levels of SARS-CoV-2 RNAs declined 72 h later in all cell lines (Figure 1B). Thus, in spite of detectable levels of ACE-2 on their cell surface, both myeloid cell lines do not seem to sustain productive SARS-CoV-2 infection, regardless of their HIV infection status, as previously observed with primary human MDM [25].

### 3.2. Modest Reversal of HIV-1 Latently Infected U1 Cells by Cell-Free SARS-CoV-2

It has been reported that, upon exposure to SARS-CoV-2, macrophages activate innate immune responses, including IFN expression, and upregulate IFN downstream pathways [29]. This phenomenon seems to be restricted to those macrophages expressing ACE2, as ACE2-negative macrophages did not release pro-inflammatory cytokines or antiviral mediators when exposed to SARS-CoV-2 [30].

**Figure 2:**
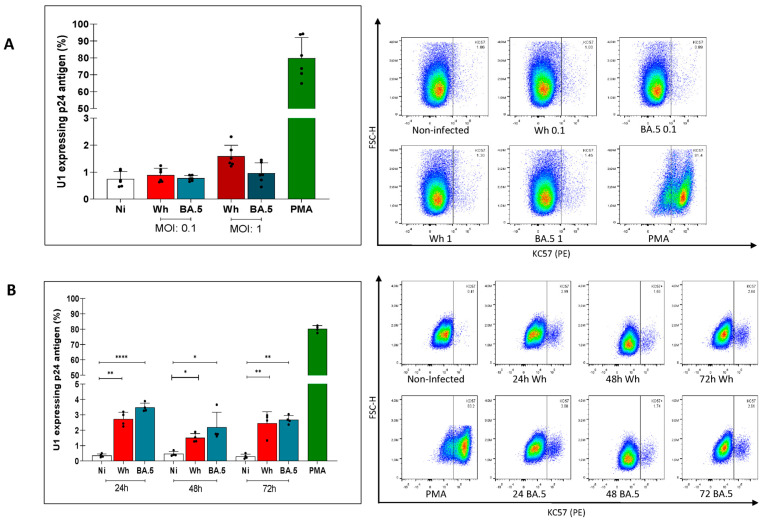
(**A**) Level of HIV latency reversal in myeloid cells exposed to two variants (Wh, BA.5) of cell-free SARS-CoV-2 for 4 h, and two inoculums (MOI: 0.1 and 1.0). (**B**) Level of HIV latency reversal in myeloid cells exposed to cell-free SARS-CoV-2 (MOI: 0.1), during extended times (24, 48, and 72 h), two variants (Wh, BA.5). Representative dot plots of flow cytometry data are showing (**right**). The figure shows representative examples in which HIV latency reversal among U1 cells was measured by intracellular detection of HIV-p24 capsid antigens using Kc-57-PE-labelled antibody in the different studied conditions. PMA: Phorbol 12-myristate 13-acetate was used a positive control for HIV latency reversal. Data are expressed as mean ± SD obtained from 4–6 independent experiments. (* *p* < 0.05; ** *p* < 0.01; **** *p* < 0.0001).

When U1 cells were shortly (4 h) exposed to cell-free SARS-CoV-2 at the MOIs of 1 and 0.1, no evidence of HIV latency reversal was obtained, irrespective of whether the ancestral (Wh) or the BA.5 variants were used (Figure 2A). Extending the time of SARS-CoV-2 (MOI: 0.1) exposure to 24, 48, and 72 h, a small, but detectable reversal of proviral latency was observed (Wh: 2.7 ± 0.5, 1.5 ± 0.5, and 2.4 ± 0.6%; BA:5: 3.5 ± 0.3, 2.2 ± 0.9, and 2.7 ± 0.5%, respectively) (Figure 2B), while cell viability was preserved in each condition (Supplementary Figure S1).

These results suggest that the interaction of the SARS-CoV-2 spike protein with ACE2 might trigger a cell signaling cascade leading to a modest HIV proviral activation in myeloid cells as a function of the duration of virus exposure.

### 3.3. Modulation of HIV Latency in U1 Cells by Soluble Factors Released by M1 or M2-Polarized MDMs

Various host factors have been found to modulate viral replication in vitro, including several cytokines. Specifically, pro-inflammatory cytokines such as tumor necrosis factor (TNF-α), interleukin-1 (IL-1), interferon-γ (IFN-γ), and IL-6 have been shown to upregulate HIV replication in various in vitro model systems. Conversely, anti-inflammatory cytokines such as IL-4, IL-10, and transforming growth factor (TGF-β) either suppressed or activated virus expression depending on the experimental conditions [31].

Primary human MDM were polarized towards M1 and M2 phenotypes by incubation with IFN-γ plus LPS and IL-4, respectively, as published [32]. The polarization state of polarized MDMs was assessed by determining the expression levels of the membrane markers CD80 and CD206 by flow cytometry and their release of pro-inflammatory cytokines (IL-1β, TNF-α, and IL-6), as determined by ELISA. CD80 expression was significantly enhanced in M1-MDMs vs. control and M2-MDMs, whereas CD206 showed the opposite pattern and improved in M2-MDMs also vs. control cells. Of interest, 50% of M1-MDMs maintained CD206 expression to levels comparable to those of unpolarized cells. Cytokine levels in culture supernatants were 281.4 ± 90.0 pg/mL (IL-1β), 30.8 ± 9.5 ng/mL (TNF-α), and 6.4 ± 2.4 ng/mL (IL-6), respectively, for M1-MDMs, whereas they were 55.1 ± 16.3 pg/mL (IL-1β), 1.4 ± 0.4 ng/mL (TNF-α), and 1.0 ± 0.4 ng/mL (IL-6), respectively, in M2-MDMs (Supplementary Figure S2).

We next investigated whether CM from unpolarized and M1/M2-polarized MDM could influence the state of proviral latency of U1 cells; U1 cells stimulated with PMA represented the positive control. The CM from M1-MDMs collected 24 h after cell polarization reactivated HIV expression in U1 cells in a concentration-dependent fashion. Latency reversal occurred in 38.1 ± 7.6% and 7.0 ± 3.0% of the cells with CM diluted 1:100 and 1:1000, respectively.

**Figure 3:**
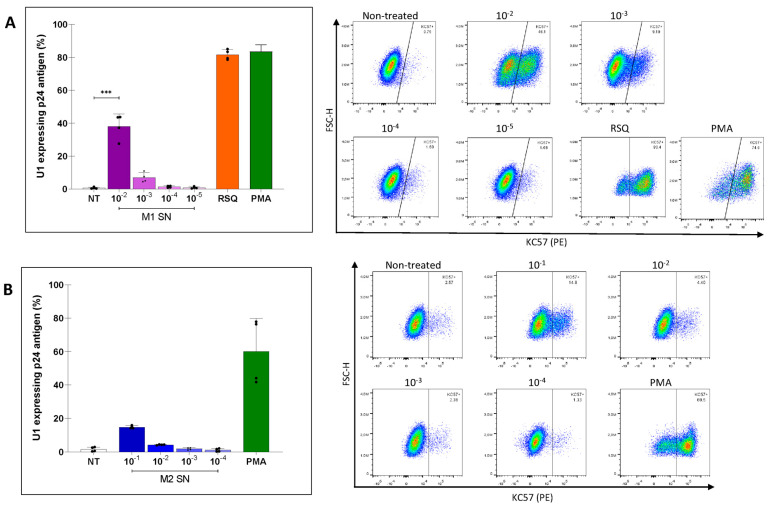
(**A**) Level of HIV latency reversal in myeloid cells exposed to conditioned media (diluted) from M1-polarized macrophages. (**B**) Level of HIV latency reversal in myeloid cells to conditioned media (diluted) from M2-polarized macrophages. Representative dot plots of flow cytometry data. The figure shows representative examples in which HIV latency reversal among U1 cells was measured by intracellular detection of HIV-p24 capsid antigens using Kc-57-PE-labelled antibody in different studied conditions (**right**). RSQ: resiquimod (R848). PMA: Phorbol 12-myristate 13-acetate was used as a positive control for HIV latency reversal. Data are expressed as mean ± SD obtained from 4 independent experiments. (*** *p* < 0.001).

We also investigated the potential effect of the imidazoquinoline Toll-like receptors (TLR) 7/8 agonist R848 (or resiquimod) that has been shown to be capable of repolarizing M2 macrophages to M1 [33]. After titration, R848 was used 1:10 vol:vol, and a strong latency reversal was observed (82.4 ± 2.4%) (Figure 3A). Furthermore, also the CM from M2-MDMs moderately reactivated HIV expression in U1 cells (14.8 ± 0.8% and 4.3 ± 0.2% for 1:10, and 1:100 dilutions, respectively), albeit to a lesser extent than that of M1-MDMs (Figure 3B). Cell viability was preserved for each condition (Supplementary Figure S3).

### 3.4. Soluble Factors Released from SARS-CoV-2-Exposed MDMs Reactivated Proviral Expression in U1 Cells

SARS-CoV-2 infection has been shown to trigger a cascade of inflammatory pathways and increase the inflammatory profiles of M1 and M0 macrophages, but not M2 macrophages [34]. A shift from M2 to M1 macrophages has been reported to be triggered by high levels of viral RNA, which serve as pathogen-associated molecular patterns (PAMPs), along with other contributing factors [18].

**Figure 4:**
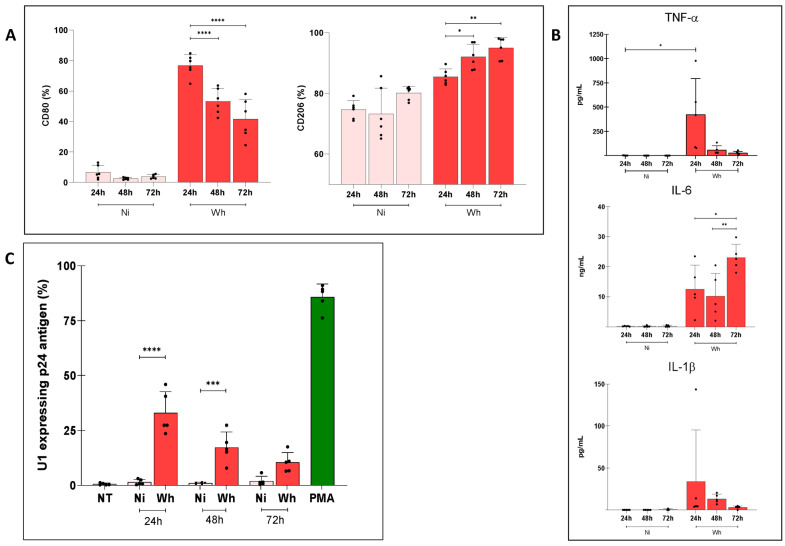
(**A**) Polarization of primary human macrophages exposed to SARS-CoV-2 (Wh) during a 4 h (short time) but with increasing post-exposure resting time (24, 48, 72 h). The upper panel shows the membrane expression levels of CD80 and CD206, expressed as a percentage on the left. (**B**) Levels of pro-inflammatory cytokines IL-1β, IL-6, and TNF-α in culture supernatants, measured by ELISA. These cell culture supernatants from each were collected to be used as “conditioned media” (CM). Ni: non-infected MDM controls. (**C**) Level of HIV latency reversal in both myeloid (U1) cells exposed to conditioned media obtained from SARS-CoV-2-short-exposed macrophages with different resting times (24, 48, 72 h). NT: non-treated; PMA: Phorbol 12-myristate 13-acetate was used as a positive control for HIV latency reversal. Data are expressed as mean ± SD from 4–6 independent experiments. (* *p* < 0.05; ** *p* < 0.01; *** *p* < 0.001; **** *p* < 0.0001).

A short-term (4 h) exposure of human M0 to SARS-CoV-2 (Wh variant) induced an M1 profile (determined by CD80 expression) 24 h post-exposure (76.8 ± 7.1%) that, however, progressively shifted towards an M2 profile (determined by CD206 expression) by 72 h post-exposure (95.0 ± 3.5%) (Figure 4A). This pattern was partially corroborated by the secretion of pro-inflammatory cytokines, which were lower than those observed by standard M1/M2 polarization, as shown in Figure 4B. The levels of cytokines measured in these supernatants at 24, 48, and 72 h were (i) TNF-α: 422.3 ± 373.0, 57.0 ± 45.0, and 25.8 ± 18.7 pg/mL, (ii) IL-1β: 34.0 ± 61.4, 13.3 ± 5.5, and 3.0 ± 1.8 pg/mL, and (iii) IL-6: 12.5 ± 8.0, 10.1 ± 7.7, 23.0 ± 4.4 pg/mL. Thus, even the highest average TNF-α concentration was 75 times lower than that previously measured in M1-MDM supernatants, while that of IL-1β was 8 times lower. Of interest, this M1/M2 shift overlapped with a diminished capacity to reactivate HIV expression in U1 cells (Figure 4C). The HIV latency reversal mediated by soluble mediators released by MDMs exposed to short-term (4 h) SARS-CoV-2 and rested for 72 h was inversely related to U1 incubation time (Figure 4C). Cell viability was preserved for each condition (Supplementary Figure S4).

Thus, SARS-CoV-2 exposure to M0 seems capable of driving their transient polarization firstly as M1 cells and then as M2 cells. This phenomenon was paralleled by a progressive loss of soluble factors reactivating proviral latency in U1 cells, particularly from M1-MDMs.

### 3.5. U1 Cells Exposed to Soluble Factors from Infectious and UV-Inactivated SARS-CoV-2-Exposed MDMs over Prolonged Time

**Figure 5:**
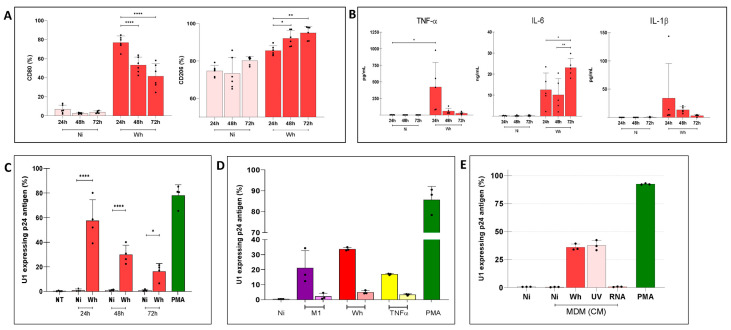
(**A**) Polarization of primary human macrophages exposed to SARS-CoV-2 during extended times (24, 48, 72). In the upper panel, the membrane expression levels of CD80 and CD206 are shown, measured by flow cytometry and expressed as percentages on the left. (**B**) The level of pro-inflammatory cytokines IL-1β, IL-6, and TNF-α in culture supernatants was measured by ELISA. These cell culture supernatants from each were collected to be used as “conditioned media” (CM). (**C**) Level of HIV latency reversal in myeloid cells exposed for a fixed time (48 h) to conditioned media collected from cultured macrophages exposed to SARS-CoV-2 during extended times (24, 48, 72 h). (**D**) TNF-α neutralization assay (right column for each condition) expressing the level of HIV latency reversal in myeloid cells exposed for a fixed time (48 h) to conditioned media collected from cultured macrophages polarized to M1 (M1), exposed to viable SARS-CoV-2 (Wh), and treated with soluble TNF-α (1 ng/mL). (**E**) Level of HIV latency reversal in myeloid cells exposed for a fixed time (48 h) to conditioned media collected from cultured macrophages exposed to viable SARS-CoV-2 (Wh), UV-inactivated virus (UV), and pure SARS-CoV-2 RNA (RNA) during 24 h. Ni: non-infected MDM controls; PMA: Phorbol 12-myristate 13-acetate was used as a positive control for HIV latency reversal. Data are expressed as mean ± SD obtained from 4–6 independent experiments. (*: *p* < 0.05; **: *p* < 0.01; ****: *p* < 0.0001).

The duration of exposure to external stimuli is a determinant of the polarization state of macrophages [35,36]. Because the severity of COVID-19 is closely related to the amount and duration of SARS-CoV-2 exposure [37], we investigated the potential relationship between variations in M1/M2 MDM polarization profiles and the viral exposure time. The polarization dynamic of MDMs following long-term (24, 48, and 72 h) exposure to SARS-CoV-2 (Wh variant) is shown in Figure 5A. Hence, as the exposure time extended from 24 to 48 to 72 h, the initial greater relative abundance of the M1 profile declined significantly from 75.5 ± 9.2% to 68.1 ± 11.5% and 51.6 ± 5.4%, respectively. Conversely, a concomitant increase of an M2 profile was observed as the virus exposure time prolonged from 64.2 ± 13.7% to 83.6 ± 6.2% and 92.0 ± 4.1%.

Among the pro-inflammatory cytokines released, TNF-α concentrations in CM mirrored the dynamics of the M1 profile: 760.4 ± 238.4, 368.5 ± 102.9, and 228.7 ± 226.7 pg/mL. Regarding IL-1β, its levels were slightly higher for extended frame times, while IL-6 concentrations did not show significant differences (Figure 5B). As shown in Figure 5C, the capacity of these three CM to reverse HIV latency in myeloid cells was indirectly correlated to the duration of exposure of MDMs to SARS-CoV-2. Thus, media obtained from MDMs exposed for 24 h showed the highest reversal capacity (57.4 ± 17.1%), followed by those obtained at 48 h (29.9 ± 7.6%), and lastly, by those collected after 72 h (16.2 ± 6.2%). The pivotal role in HIV latency reversal of TNF-α-released from SARS-CoV-2-infected macrophages was demonstrated using a neutralization assay with the human mAb Infliximab. The capacity of the CM to HIV latency reversal in U1 cells significantly decreased by 85% (from 33.7 ± 0.4 to 4.9 ± 0.3%). Cell viability was preserved in each condition (Supplementary Figure S4).

As the UV inactivation of SARS-CoV-2 enables the acquisition of viral material that retains antigenic and immunogenic properties similar to those of the native antigen as well as viral particle morphology remained intact [38,39], to elucidate whether PAMPs within the SARS-CoV-2 particle can trigger innate immune activation, we exposed MDMs to either replicative or UV-inactivated SARS-CoV-2 at the MOI of 0.1 for 24 h. The CM collected 48 h after MDM exposure to UV-inactivated virus promoted U1 latency reversal, like that observed in cells incubated with the replicative virus, without altering cell viability (Supplementary Figure S5). Their CM contained comparable concentrations of pro-inflammatory cytokines, such as TNF-α (5.1 ± 2.3 ng/mL), IL-1β (10.4 ± 4.8 pg/mL), and IL-6 (2.2 ± 0.4 ng/mL), similar to those released when using replicative SARS-CoV-2. This suggests that SARS-CoV-2-derived PAMPs can promote soluble mediators release as part of the innate immune activation of macrophages, independent of viral replication. In contrast, soluble SARS-CoV-2 RNA was unable to promote any detectable MDM response that prompted HIV latency reactivation in U1 cells (Figure 5E).

## 4. Discussion

In the present study, we have investigated the potential interplay between SARS-CoV-2 and HIV-1 in myeloid cells. Although incubation of primary human MDMs with SARS-CoV-2 did not result in productive infection, nonetheless, the virus was sensed and triggered a pro-inflammatory response with release of related cytokines. This was particularly observed if MDMs had been previously polarized as M1 cells by stimulation with LPS and IFN-γ and, to a lower extent, as M2 cells by exposure to IL-4. The chronically HIV-infected myeloid cell line U1, a model of reversible proviral latency, was used to investigate the potential impact of different experimental conditions on virus expression. Indeed, CM from M1-MDMs and from SARS-CoV-2 MDMs reactivated proviral latency in U1 cells, whereas SARS-CoV-2 induced a progressive shift of MDM profile from an initial M1 to an M2 profile that was reflected in a progressively diminishing capacity of inducing HIV expression in U1 cells.

The disruptions in immune function and inflammation induced by SARS-CoV-2 may have lasting effects on HIV dynamics, potentially extending beyond acute infection. Our study suggests that SARS-CoV-2 can indeed reverse HIV latency at least in infected myeloid cells. Such reactivation occurs to a lesser extent through direct virus–cell interaction but mainly through an indirect bystander mechanism involving SARS-CoV-2-exposed macrophages releasing pro-inflammatory cytokines. Myeloid cells play a role in HIV latency within specific tissues and organs, such as the central nervous system, along with CD4+ T cells, which are latently infected and harbor integrated, replication-competent provirus [40]. Various in vitro models of HIV latency have been established over time, encompassing latently infected cell lines and primary T-cells such as the U1 model [41,42,43], in which canonical NF-κB activation, among other transcription factors, leads to the activation of HIV transcription and reversal of latency [11].

Angiotensin-converting enzyme 2 (ACE2) is the primary entry receptor for SARS-CoV-2 [44], and its expression can be induced by in vivo viral infection or in vitro IFN stimulation [45]. Indeed, ACE2 is an IFN-stimulated gene (ISG) whose levels strongly correlate with ISG-induced NF-κB activation [46]. Here, we report that the basal expression levels of ACE2 in the myeloid cell model of HIV latency U1 were as low as those of their uninfected counterpart, the U937 promonocytic cell line. Consistently with previous reports on primary MDMs, SARS-CoV-2 incubation with both cell lines did not result in detectable virus replication. However, this abortive infection did not preclude interaction with ACE2 that could activate intracellular signaling cascades ultimately promoting cellular activation [25,47]. Indeed, when U1 cells were exposed to cell-free SARS-CoV-2 or M1-MDM-derived CM, ACE2 expression was upregulated. Reversal of HIV latency in U1 cells through direct interaction with cell-free SARS-CoV-2 occurred, but it required extended exposure periods (≥24 h) to achieve a low level of reactivation (<4%), regardless of whether the ancestral or Omicron SARS-CoV-2 variants were used as inoculum. This effect could be related to the induction of autocrine loops involving the release of endogenous TNF-α and IL-1β, contributing to the reversal of HIV latency during prolonged exposure, beyond the ACE2–virus interaction [48].

Macrophages exhibit high plasticity, allowing for the distinction between two main subtypes: classically activated, pro-inflammatory M1 macrophages and alternatively activated, anti-inflammatory M2 macrophages. M1 macrophages produce pro-inflammatory cytokines, such as TNFα, IL-1β, and IL-6, while M2 macrophages produce IL-10, TGF-β, and CCL18. The frequent presence of hybrid phenotypes lacking clear M1 or M2 markers makes the M1/M2 paradigm appear overly simplistic, but it remains a useful paradigm for many experimental investigations [49]. A previous report showed that MDMs, which replace alveolar macrophages and dominate macrophage lineages in severely damaged lungs, are highly inflammatory and potent cytokine producers [50]. Moreover, among COVID-19 patients, under acute disease progression, monocytes differentiate into MDMs while still in circulation; this newly identified population of massively activated circulating monocytes/macrophages produces high levels of TNF-α, IL-6, and IL-10, potentially contributing to the cytokine storm [51]. We and others have reported that the interaction between SARS-CoV-2 and ACE2 in MDMs triggers signals that regulate their activation, influencing the production of these pro-inflammatory cytokines and chemokines [25,52].

Here, we investigated the distinct abilities of soluble mediators released by MDMs in vitro, depending on their M1 or M2 profiles, to promote a significant reversal of HIV latency in U1 cells. Mediators secreted by M1-MDMs, mainly TNF-α, induced latency reversal, whereas those released by M2-MDMs showed a significantly lower efficiency. Additionally, other soluble factors released by M2-MDMs, such as TGF-β and IL-4, have been reported to inhibit the reactivation of latent HIV [53,54].

Notably, the M1/M2 profiles after in vitro M0-MDM–SARS-CoV-2 interaction varied according to the viral stimulus duration and the subsequent resting time. Firstly, MDMs briefly exposed (4 h) to SARS-CoV-2 followed by 24 h of resting depicted a prominent M1 polarization and released high levels of TNF-α and IL-1β. Afterward, when resting times were longer, a dynamic change in macrophage activation was observed, switching toward an M2 profile associated with smoldering the microenvironment inflammation; this swapping was accompanied by a diminished HIV latency reversal level in U1 cells. Second, when MDMs were exposed to SARS-CoV-2 for extended periods (≥24 h), the varying levels of pro-inflammatory cytokines—particularly TNFα—once again determined the ability of the conditioned media to reverse HIV latency in U1 cells. M1 predominance, characterized by higher levels of TNF-α, was observed after 24 h exposure to SARS-CoV-2, which then shifted to M2 with lower TNF-α levels for longer (48–72 h) virus exposures.

Although the duration of the replication cycle of SARS-CoV-2 in ranges from 7 to 24 h [55], virus-infected cells release viral proteins into the extracellular media relatively early in the infection process, usually within 6 to 12 h post-infection [56]. These viral proteins might also function as virokines, modulating the monocyte/macrophages activation status and influencing the reactivation of HIV latently infected cells. Accordingly, we observed that UV-inactivated SARS-CoV-2, which preserves protein integrity, promotes similar levels of latency reversal in U1 cells as the replicative virus. This finding reinforces the relevance of bystander innate immune-mediated HIV reactivation beyond SARS-CoV-2 replication.

This study has some limitations. Firstly, the biology of the U1 cell line may not accurately represent that of resting infected macrophages—including TRMs—which serve as reservoirs for latent HIV-1 in vivo, as the mechanisms controlling latency and reactivation in cell lines can differ substantially from those in primary cells [57]. Secondly, the absence of the microenvironment context, including cell-to-cell interactions and the extracellular matrix, limits the ability to study how these factors influence HIV latency. Thirdly, we did not investigate the potential, direct, and indirect effects of SARS-CoV-2 and the potential role of macrophages on latently infected CD4+ T lymphocytes or lymphocytic cell lines.

## 5. Conclusions

This study provides experimental evidence that SARS-CoV-2, either directly or through factors released by macrophages interacting with SARS-CoV-2, can reverse HIV latency in infected myeloid cells, as here shown for U1 cells. The interplay between SARS-CoV-2 infection and HIV latency is complex and likely involves multiple cellular pathways and immune responses, including the expression of ACE2 and the abundance of pro-inflammatory soluble mediators, such as TNF-α. Therefore, more research is needed to fully elucidate the exact mechanisms and the extent to which SARS-CoV-2 can influence the state of HIV latency. These findings require further investigation, as well as addressing their implications for people living with HIV.

## Data Availability

The original contributions presented in the study are included in the article/supplementary material, further inquiries can be directed to the corresponding author.

## Supplementary Materials

The following supporting information can be downloaded at: https://www.mdpi.com/article/10.3390/v16081310/s1, Figure S1: Cell death rate among U1 and U937 cells exposed to cell-free SARS-CoV-2; Figure S2: (A) Characterization of primary human macrophages polarized with LPS and INF-γ (M1) and with IL-4 (M2). (B) Levels of pro-inflammatory cytokines IL-1β, IL-6, and TNF-α in culture supernatants were quantified by ELISA.; Figure S3: Cell death rate in U1 cells exposed to conditioned media from M1 (left) and M2 (right) polarized macrophages; Figure S4: Cell death rate in U1 cells exposed to conditioned media from SARS-CoV-2 exposed MDM for a short time and collected at different resting times (left), and prolonged-times (right); Figure S5: Level of HIV-latency reversal and cell death rate in myeloid cells exposed for a fixed time (48 h) to different TNF-α concentrations (left).
