# Supplementary figures and images for: SARS-CoV-2 Modulation of HIV Latency Reversal in a Myeloid Cell Line: Direct and Bystander Effects
Source: Viruses. 2024 Aug 17;16(8):1310. doi: 10.3390/v16081310 (PMC11359691; doi:10.3390/v16081310)

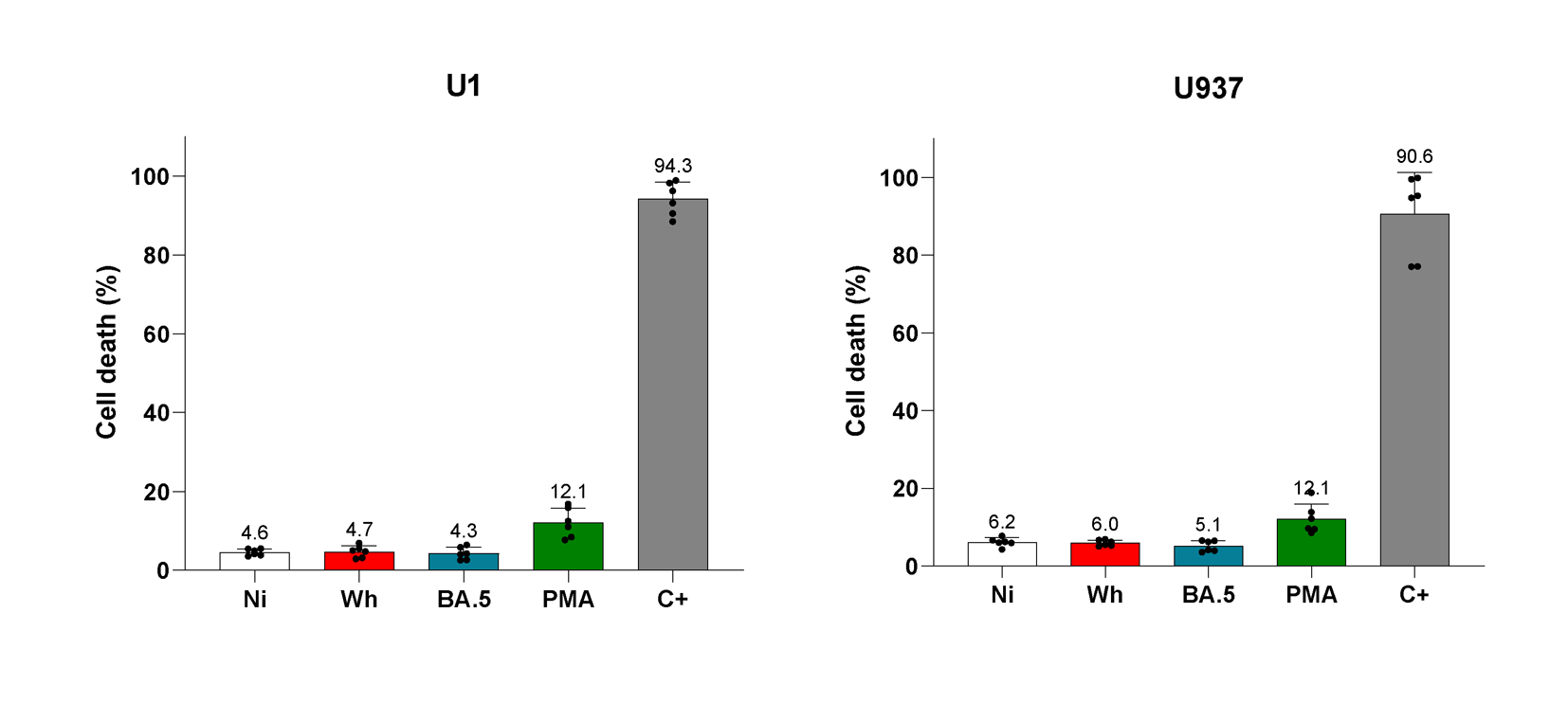

Supplement: Supplementary file 1 [file viruses-16-01310-s001.zip › SuppFig1.tiff]

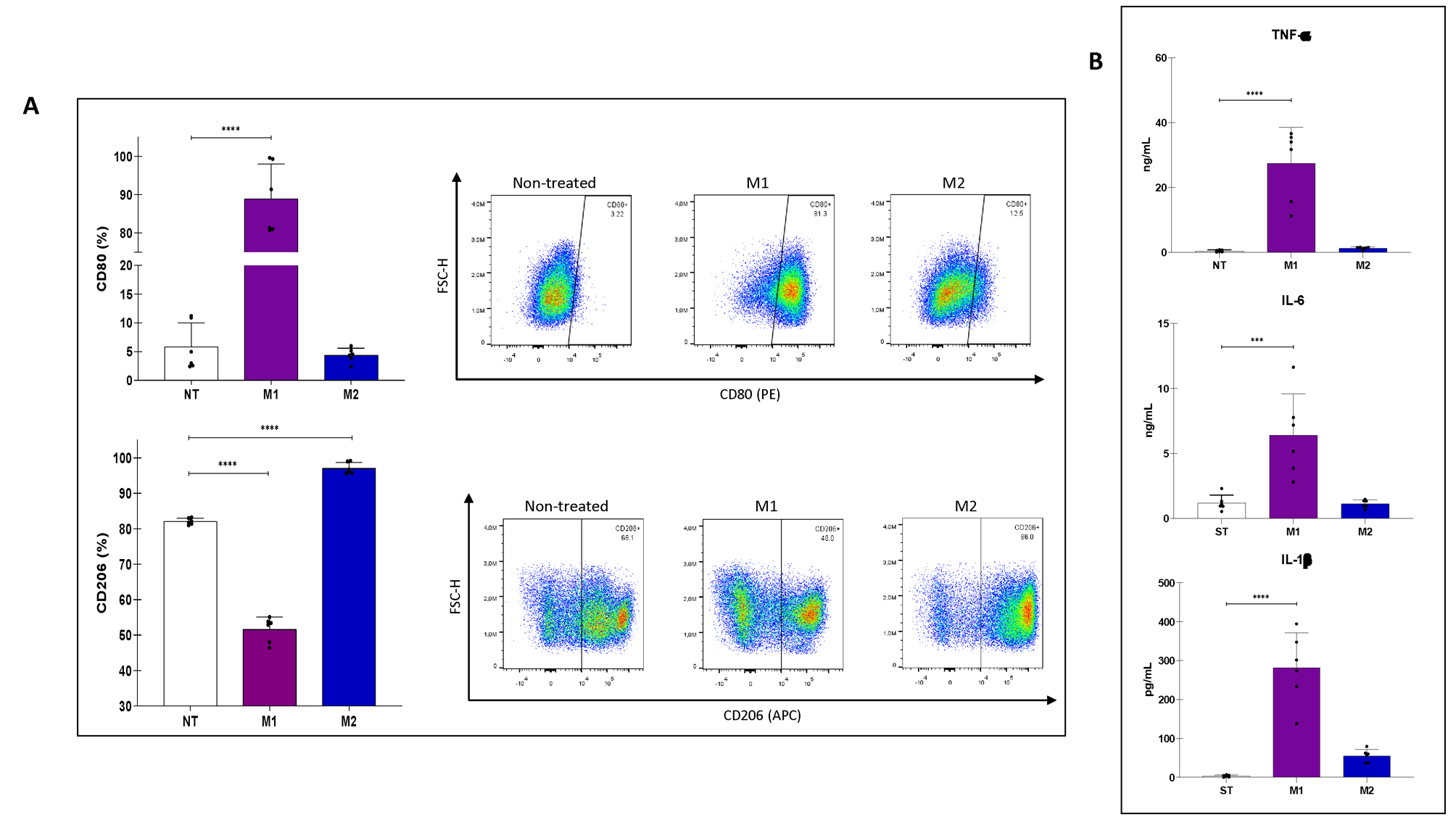

Supplement: Supplementary file 1 [file viruses-16-01310-s001.zip › SuppFig2.tiff]

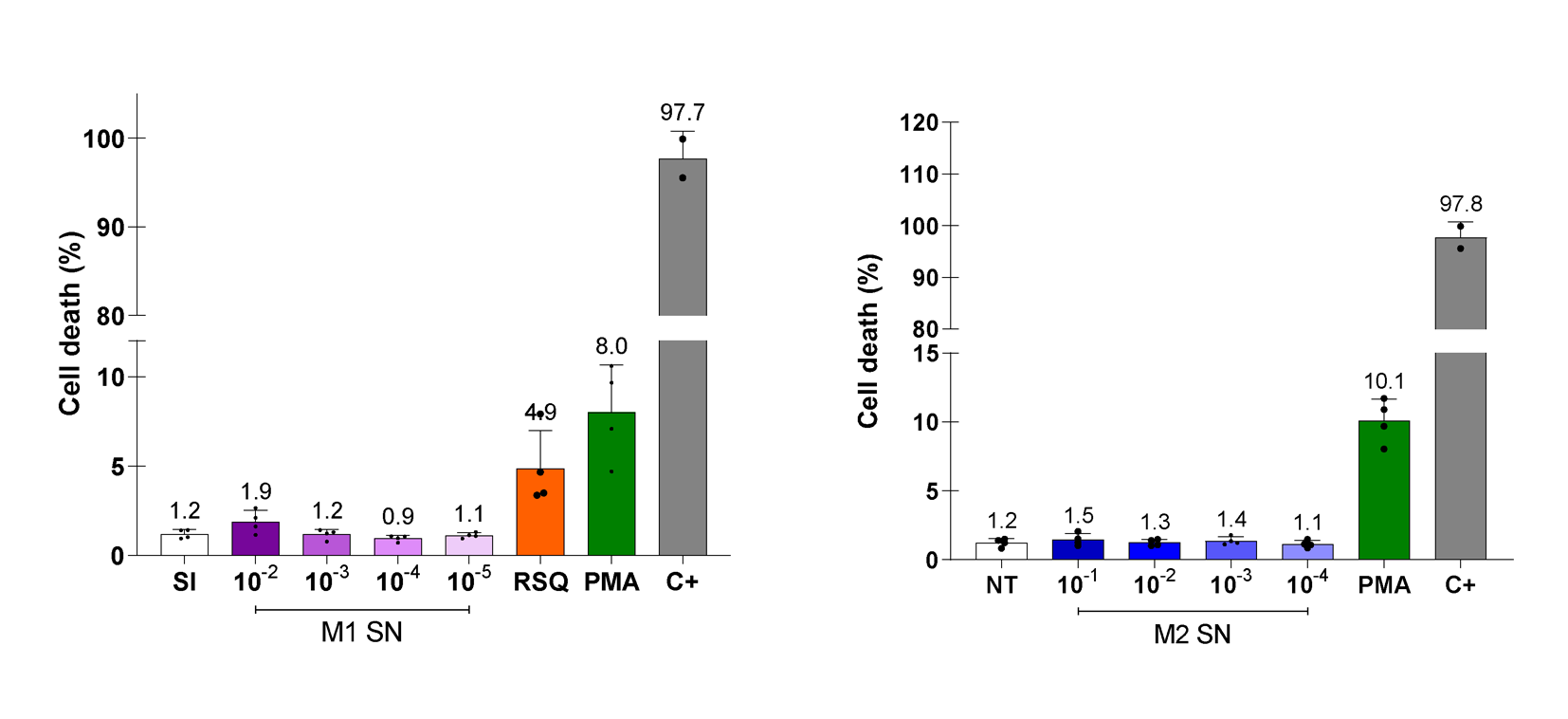

Supplement: Supplementary file 1 [file viruses-16-01310-s001.zip › SuppFig3.tiff]

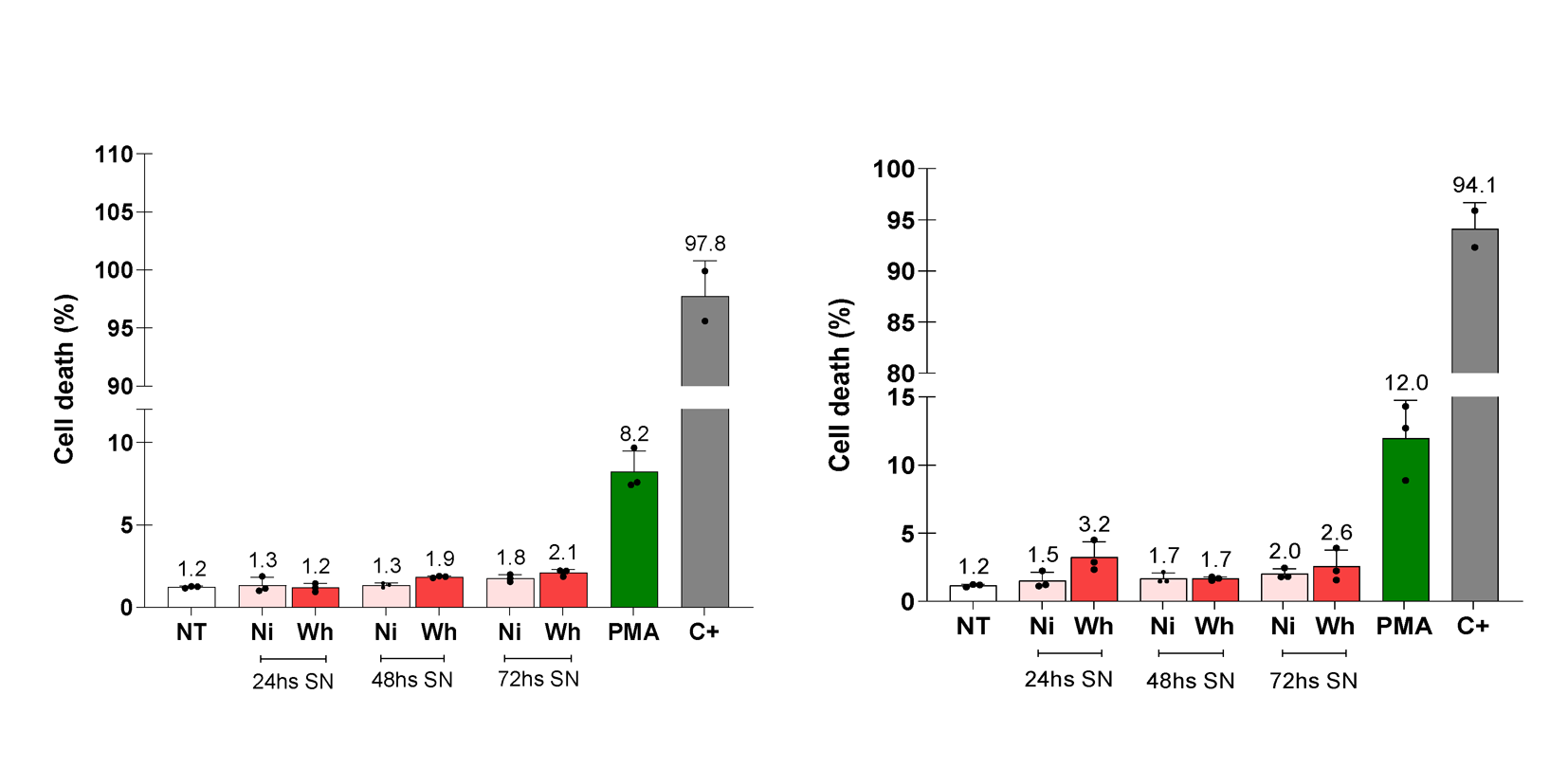

Supplement: Supplementary file 1 [file viruses-16-01310-s001.zip › SuppFig4.tiff]

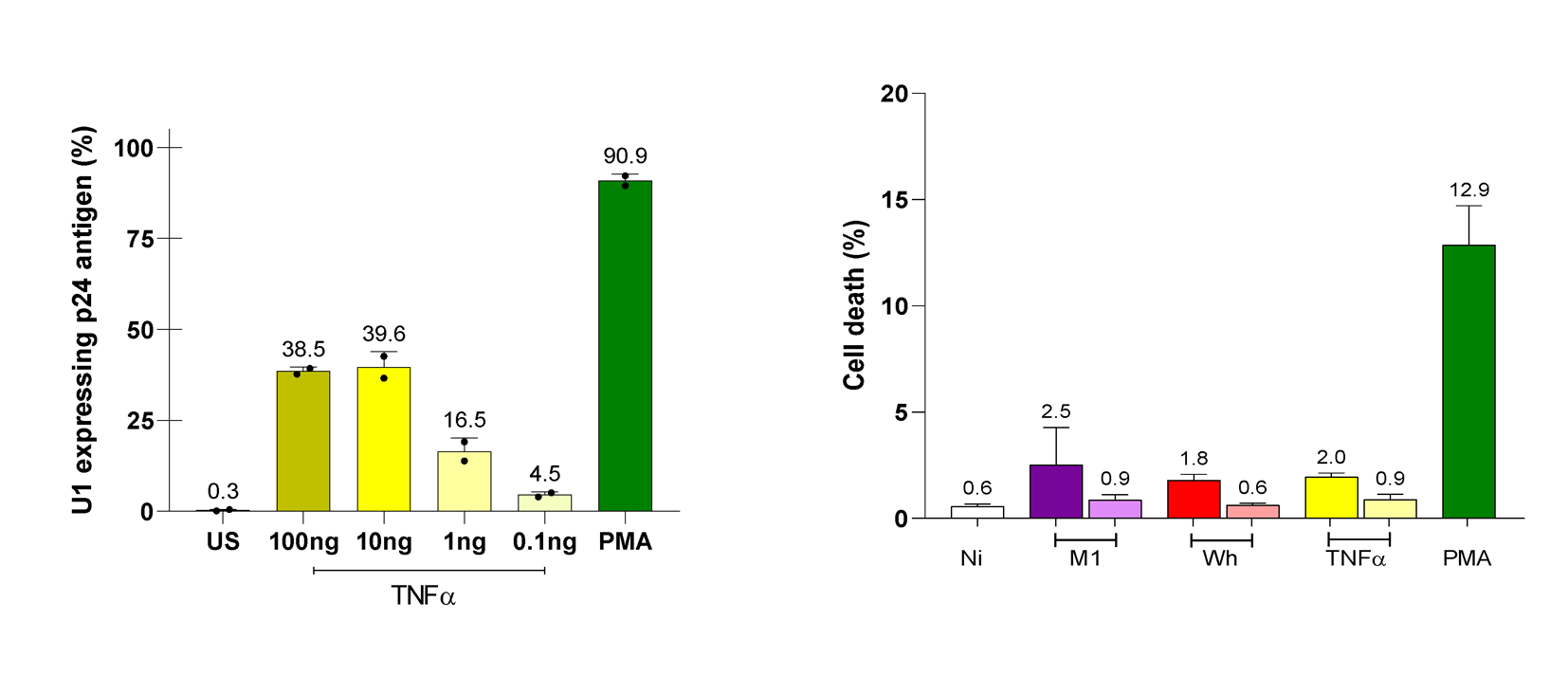

Supplement: Supplementary file 1 [file viruses-16-01310-s001.zip › SuppFig5.tiff]
